# Anti-Tumor Activity and Immunotherapeutic Potential of a Bisphosphonate Prodrug

**DOI:** 10.1038/s41598-017-05553-0

**Published:** 2017-07-20

**Authors:** Yoshimasa Tanaka, Masashi Iwasaki, Kaoru Murata-Hirai, Kenji Matsumoto, Kosuke Hayashi, Haruki Okamura, Tomoharu Sugie, Nagahiro Minato, Craig T. Morita, Masakazu Toi

**Affiliations:** 1 Center for Innovation in Immunoregulative Technology and Therapeutics, Graduate School of Medicine, Kyoto University, Yoshidakonoe-cho, Sakyo-ku Kyoto, 606-8501 Japan; 2 Department of Immunology and Cell Biology, Graduate School of Medicine, Kyoto University, Yoshidakonoe-cho, Sakyo-ku, Kyoto 606-8501 Japan; 3 Center for Bioinformatics and Molecular Medicine, Graduate School of Biomedical Sciences, Nagasaki University, 1-12-4 Sakamoto, Nagasaki, 852-8523 Japan; 4 Department of Tumor Immunology and Cell Therapy, Hyogo College of Medicine, 1-1 Mukogawa-cho, Nishinomiya, Hyogo 663-8501 Japan; 5 Department of Surgery, Graduate School of Medicine, Kyoto University, 54 Kawahara-cho, Shogoin, Sakyo-ku, Kyoto 606-8507 Japan; 6 Department of Internal Medicine and the Interdisciplinary Graduate Program in Immunology, University of Iowa Carver College of Medicine, Veterans Affairs Health Care System, 601 Highway 6 West, Research (151), Iowa City, IA 52246 USA

## Abstract

Bisphosphonates have benefits in breast cancer and multiple myeloma patients and have been used with adoptive immunotherapy with γδ T cells expressing Vγ2 Vδ2 TCRs. Although treatment with γδ T cells is safe, it has shown limited efficacy. Present bisphosphonates stimulate γδ T cells but were designed to inhibit bone resorption rather than treating cancer and have limited oral absorption, tumor cell entry, and cause bone side effects. The development of phosphate and phosphonate nucleotide prodrugs has led to important drugs for hepatitis C and HIV. Using a similar approach, we synthesized bisphosphonate prodrugs and found that they efficiently limit tumor cell growth. Pivoxil bisphosphonate esters enter cells where esterases convert them to their active acids. The bisphosphonate esters stimulated γδ T cells to secrete TNF-α in response to a variety of tumor cells more efficiently than their corresponding acids. The most active compound, tetrakis-pivaloyloxymethyl 2-(thiazole-2-ylamino)ethylidene-1,1- bisphosphonate (**7**), specifically expanded γδ T cells and stimulated them to secrete interferon-γ and kill tumor cells. In preclinical studies, combination therapy with compound **7** and γδ T cells prolonged survival of mice inoculated with either human bladder cancer or fibrosarcoma cells. Therefore, bisphosphonate prodrugs could enhance the effectiveness of adoptive cancer immunotherapy with γδ T cells.

## Introduction

Geminal bisphosphonates with P-C-P structure were initially developed as anti-corrosion agents for industrial purposes^1^. The first-generation bisphosphonates such as etidronate and clodronate show high affinity for bone minerals and are metabolized to cytotoxic β, γ-methylene analogs of ATP in osteoclasts^2^. Extensive synthetic efforts resulted in second-generation bisphosphonates with alkylamine side chains and third-generation bisphosphonates with nitrogen-containing heterocyclic side chains for the treatment of patients with osteoporosis and hypercalcemia of malignancy^3^.

These nitrogen-containing bisphosphonates enter monocyte-lineage cells (including osteoclasts) as well as tumor cells where they inhibit farnesyl diphosphate synthase (FDPS)^4, 5^. FDPS inhibition reduces the levels of its downstream metabolites, farnesyl diphosphate (FPP) and geranylgeranyl diphosphate (GGPP), and leads to the production of a toxic ATP analog, triphosphoric acid 1-adenosin-5′-yl ester 3-(3-methylbut-3-enyl) ester (ApppI)^6^. The loss of FDPS metabolites impairs their transfer to the C-termini of small GTPases, such as RAS, RAP, RHO, and RAB, and the γ subunit of G protein-coupled receptors, that is required for their functions in signal transduction and cell survival.

Inhibition of FDPS also leads to the accumulation of its upstream metabolite, isopentenyl pyrophosphate (diphosphate) (IPP)^7–9^, which in turn stimulates cytotoxic γδ T cells through their Vγ2 Vδ2 TCRs by the sensing of IPP binding to the B30.2 domain of butyrophilin (BTN) 3A1^10–14^. These activated γδ T cells exhibit potent cytotoxic activity against bisphosphonates-pulsed tumor cells^15^.

The addition of intravenous zoledronic acid to standard therapies improves disease-free survival of breast cancer patients in a low estrogen environment^16–18^ and overall survival of multiple myeloma patients^19^ and lung cancer patients^20^. The use of oral bisphosphonates (alendronate or risedronate) also improves survival of breast cancer patients^21^. The anti-tumor activity of bisphosphonates is likely mediated through several pathways; one possible pathway is by promoting the anti-tumor activity of γδ T cells.

γδ T cells bearing Vγ2 Vδ2 TCRs have been targeted for cancer immunotherapy in two ways. In one approach, Vγ2Vδ2 T cells are directly stimulated *in vivo* either by an IPP analog or an aminobisphosphonates in conjunction with IL-2^22–26^. This approach has had limited success because expansion of Vγ2Vδ2 T cell does not always occur and because deletion and/or anergy of Vγ2Vδ2 T cells rapidly develops. A second approach has been the *ex vivo* expansion of Vγ2Vδ2 T cells followed by their adoptive transfer^27–36^. This approach avoids the development of anergy by harvesting and freezing of PBMC prior to therapy or by avoiding the use of intravenous bisphosphonates. However, the most successful clinical trials have used intravenous zoledronic acid around the time of adoptive transfer of Vγ2Vδ2 T cells.

One potential way to improve γδ T cell immunotherapy is to increase the potency of the bisphosphonate used by facilitating tumor cell entry. Oldfield and co-workers have improved the activity of bisphosphonates by incorporating long acyl chains^37, 38^ or by masking the P-C-P structure with pivoxil esters^39^. Pivoxil bisphosphonate esters have also been used to inhibit geranylgeranyl diphosphate synthase^40^. Similar masking of the negative charges of the phosphate moieties of nucleotides to make prodrugs has greatly improved their effectiveness in treating hepatitis C and human immunodeficiency virus infections^41, 42^.

**Figure 1:**
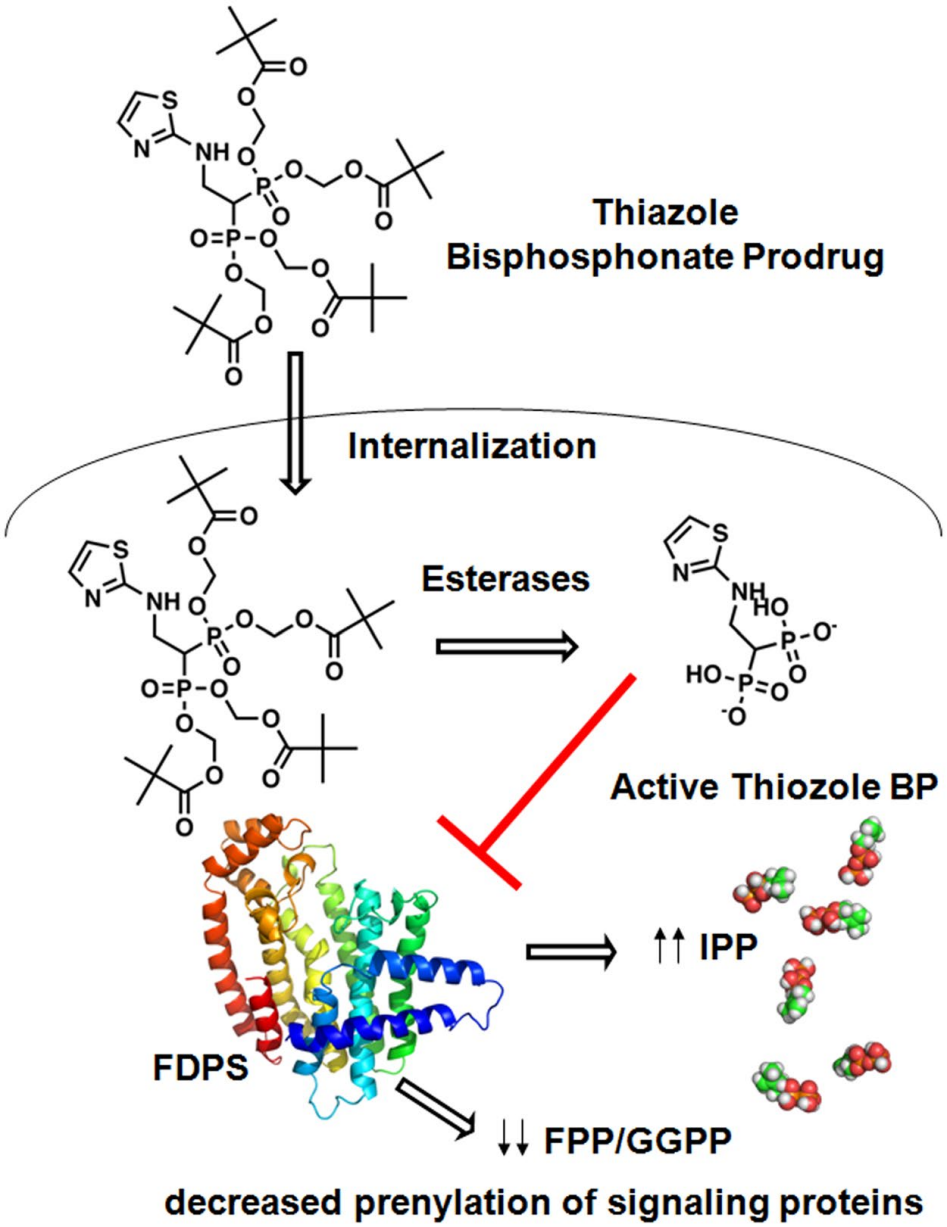
Schematic diagram of intracellular conversion of bisphosphonate pivoxil prodrugs to their corresponding acids. Bisphosphonate prodrugs are taken up by tumor cells where intracellular esterases remove their pivaloyloxymethyl moieties to yield their acid forms. The active acid forms inhibit FDPS resulting in increases in an upstream metabolite, IPP, that stimulate Vγ2Vδ2 T cells and decreases in downstream farensyl diphosphate and geranylgeranyl diphosphate resulting in decreased prenylation of signaling proteins inhibiting tumor cell growth and function.

We recently synthesized pivaloyloxymethyl (pivoxil) derivatives of bisphosphonates^43^. The resulting bisphosphonate prodrugs were efficiently internalized into tumor cells where intracellular esterases converted them into biologically active bisphosphonic acids that block FDPS. FDPS inhibition slows tumor growth through inhibition of prenylation of signal transduction molecules (schematic is shown in Fig. 1). Blocking of FDPS will also increase IPP levels in tumor cells which should make them stimulatory for Vγ2Vδ2 T cells through their Vγ2 Vδ2 TCRs.

In this study, we have assessed the ability of these bisphosphonate prodrugs to stimulate γδ T cells and to inhibit prenylation in a variety of tumor cell lines. Bisphosphonate prodrugs potently stimulated γδ T cells with pretreatment of the different cell lines. A thiazole bisphosphonate prodrug, **7**, showed the highest potency for stimulation of γδ T cells and inhibition of RAP1A prenylation. **7** specifically stimulated Vγ2Vδ2 T cells to expand from blood lymphocytes, secrete IFN-γ, and kill tumor cells. In preclinical studies, **7** combined with Vγ2Vδ2 T cells prolonged survival of immunodeficient NOG mice implanted with either human bladder cancer or fibrocarcinoma cells more than Vγ2Vδ2 T cells alone.

## Results

### Enhancement of TNF-α secretion from γδ T cells by pivaloyloxymethylation of bisphosphonates

**Figure 2:**
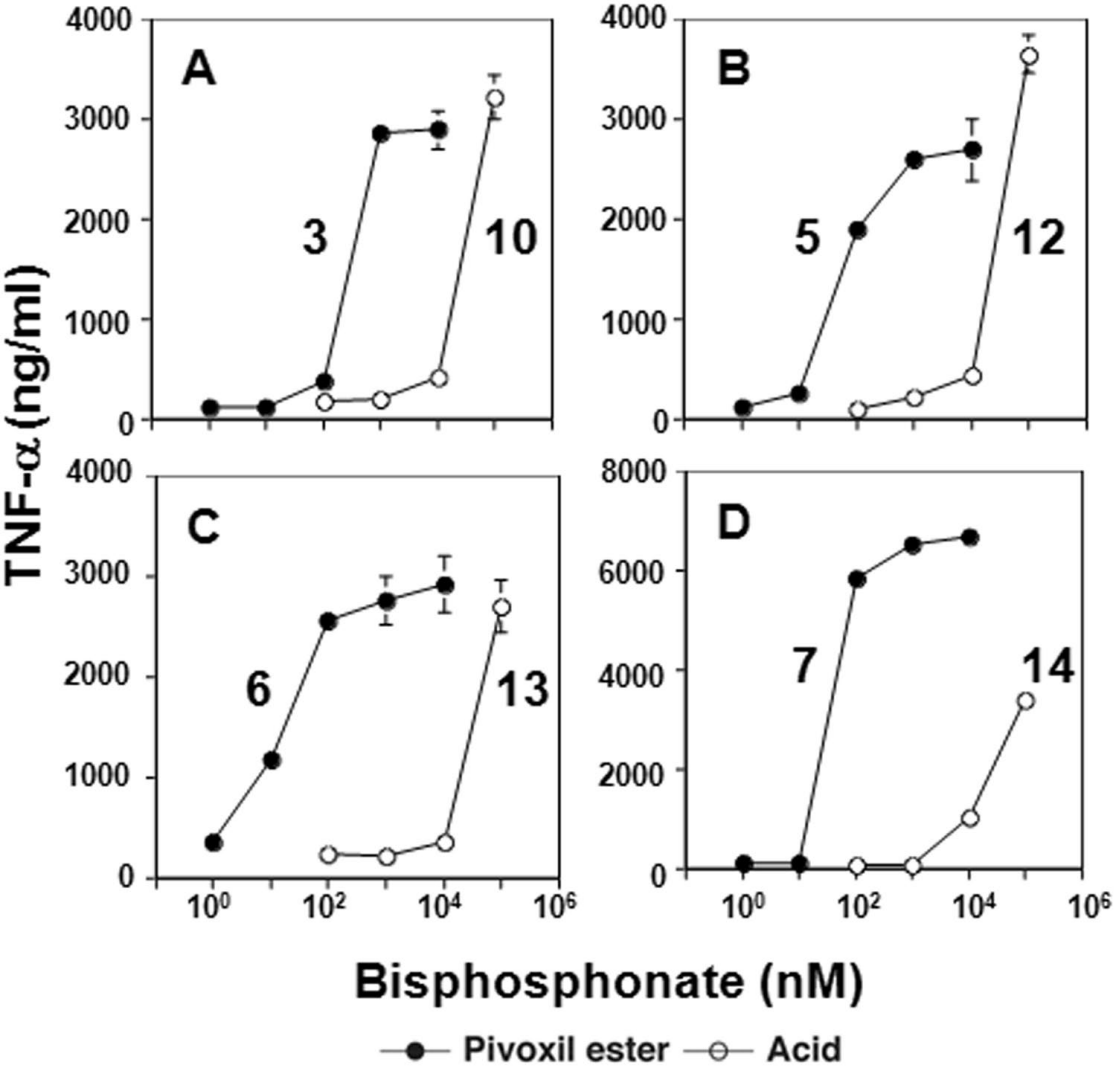
TNF-α secretion by γδ T cells stimulated with tumor cells pretreated with BP prodrugs and their acids. TNF-α production by γδ T cells in response to EJ-1 bladder cancer cells pretreated with various concentrations of bisphosphonate prodrugs was compared with that in response to EJ-1 bladder cancer cells pretreated with their corresponding acids: (**A**) compounds 3 (⚫) and 10 (⚪), (**B**) 5 (⚫) and 12 (⚪), (**C**) 6 (⚫) and 13 (⚪), (**D**) 7 (⚫) and 14 (⚪).

We previously showed that masking the phosphonate charges of bisphosphonates by pivoxil groups greatly enhanced their ability to inhibit tumor cell growth^43^. To determine if the stimulation of Vγ2Vδ2 T cells showed similar enhancement, EJ-1 bladder carcinoma cells were treated with seven bisphosphonate prodrugs or their corresponding active acid forms (Supplemental Fig. S1). Five of these pairs (**2**–**6**/**9**–**13**) were based on NE11809 (compound **8**)^44, 45^. A final compound substituted a thiazole for the pyridine group. Their synthesis has been described^43^. All of the bisphosphonate prodrugs stimulated TNF-α secretion from γδ T cells more effectively than their corresponding acid forms (Fig. 2). For example, compound **7** was 1,100-fold more active than its acid, **14** (Fig. 2D). Bisphosphonate prodrug concentrations required to stimulate half-maximal TNF-α production (EC_50_) from γδ T cells were between 20-fold to 1,100-fold lower than their acid forms (Supplemental Table S1) with several (**5**, **6**, and **7**) active at less than 80 nM. These differences were larger than the values we previously reported for growth inhibition of EJ-1 cells with eight of the nine pivoxil esters active at lower concentrations^43^.

Based on these findings, we tested 28 additional bisphosphonate pivoxil and other esters (compounds **15**–**42**, Supplemental Fig. S2) for their ability to stimulate TNF-α release by γδ T cells. Again **7** was the most potent although its methyl- derivative (**34**) had similar activity as did compound **39**, another analog of **8**, that is similar in structure and potency to **5** and **6** (Supplemental Table S2). Given that compound **7** was the most active for γδ T cell activation and tumor growth inhibition^43^, we focused on it for further study.

### γδ T cell activation by compound 7

**Figure 3:**
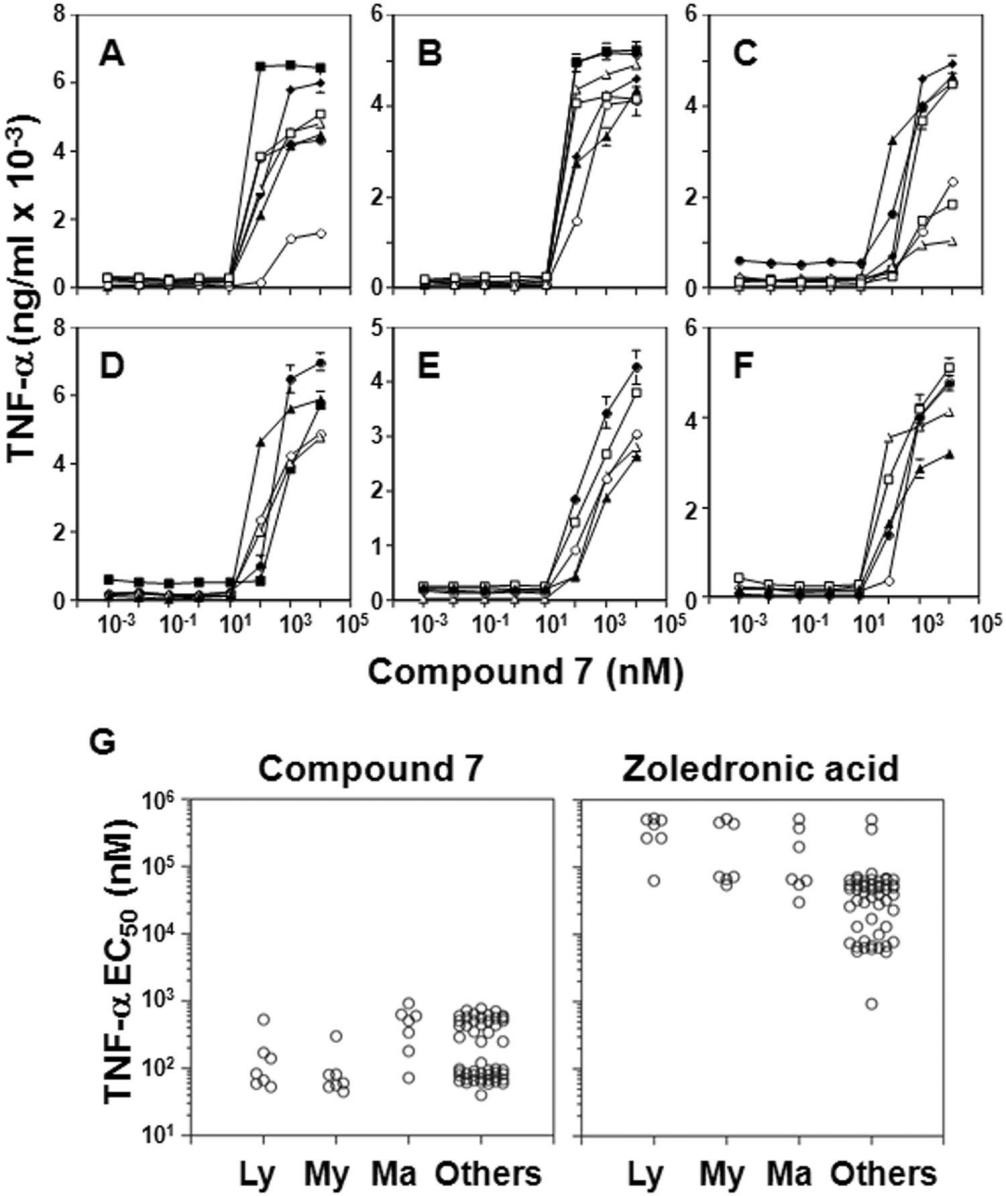
Stimulation of TNF-α secretion from γδ T cells by tumor cells pretreated with compound 7. (**A**–**F**) Stimulation of TNF-α production by γδ T cells in response to compound **7** pretreatment of various tumor cell lines; (**A**) lymphomas, ▫ MOLT-3, ∆ PEER, ⚪C1R, ⬧J.RT3-T3.5, ▪ Raji, ▴ RAMOS-RA1, ⚫MOLT-4; (**B**) myeloid leukemias, ▫ HL60, ∆ U937, ⚪THP-1, ⬧ SCC-3, ▪ P31/FUJ, ▴ K562,⚫ NOMO-1; (**C**) mammary carcinomas, ▫ YMB-1-E, ∆ MRK-nu-1, ○ HMC-1-8, ⬧ MCF-7, ▪ MDA-MB-231, ▴T-47D, ⚫ SK-BR-3; (**D**) renal cell carcinomas, ▴ 786-0, ∆ VMRC-RCZ, ⚫ UOK121, ⚪ Caki-1, ▪ A-704; (**E**) pancreatic carcinomas, ⚫ BXPC-3, ▴ KP4-1, ⚪ KP4-2, ▫ KP4-3, ∆ MiaPaCa2; (**F**) other tumor cells, ⚫ TGB24TKB cholangiocell carcinoma, ▴ ACS gastric carcinoma, ⚪ MG63 osteocarcoma, ▫ LK-2 lung carcinoma, ∆ C32TG melanoma. (**G**) Comparison of compound **7** (left panel) with zoledronic acid (right panel) concentrations used to pretreat tumor cells for half-maximal stimulation of TNF-α production by Vγ2Vδ2 T cells. Ly = lymphomas, My = myeloid leukemia, Ma = mammary carcinomas, and Others = other tumor cell lines. Details of zoledronic acid stimulation of Vγ2Vδ2 T cells are from Idrees *et al*.^46^ and are included for comparison.

**7** was also more active than its acid form, **14**, in stimulating release of TNF-α by Vγ2Vδ2 T cells upon testing an additional 21 tumor cell lines, ranging from 81-fold to 1,900-fold more potent (Table 1 and Supplemental Fig. S3). **7** was further tested against a variety of tumor cell lines in comparison to zoledronic acid, the most potent FDA-approved bisphosphonate (Fig. 3 and Supplemental Table S3). The EC_50_ values for most tumor cell lines were less than 1 μM (Fig. 3) and 76 out of 77 tumor cell lines elicited TNF-α responses by Vγ2Vδ2 T cells. The proportion of tumor cell lines with EC_50_ values of 100 nM or less was 57.1% for lymphoma, 85.7% for myeloid leukemia, 14.3% for mammary carcinoma, and 51.9% for other tumor cell lines. The average EC_50_ ± S.D. was 157 ± 170 nM for lymphomas, 96 ± 91 nM for myeloid leukemias, 465 ± 290 nM for mammary carcinomas, and 298 ± 240 nM for other tumor cell lines. These values were significantly lower than those for zoledronic acid, where the average EC_50_ ± S.D. was 367,571 ± 173,160 nM (2,337-fold greater than that of compound 7) for lymphomas, 240,571 ± 219,133 nM (2,495-fold) for myeloid leukemias, 187,771 ± 191,657 nM (404-fold) for mammary carcinomas, and 52,223 ± 82,697 nM (190-fold) for other tumor cell lines^46^. Thus, **7** is a significantly more potent activator of γδ T cells than zoledronic acid.

**Table 1:** Effect of pivoxil esterification of **14** on TNF-α secretion from γδ Τ cells stimulated with bisphosphonate-pretreated tumor cells.

Tumor cell	Origin	TNF-α EC_50_ (nM)		Ratio
		7 (PE)	14 (H)	7 EC_50_:14 EC_50_
786-0	Renal cell carcinoma	65	>100,000	1: >1,500
MKN1	Gastric cancer	560	89,000	1: 160
OST	Osteosarcoma	600	>100,000	1: >170
PC-3	Prostate cancer	65	77,000	1: 1,200
PK1	Pancreatic cancer	450	>100,000	1: >200
LK-2	Squamous NSCLC	97	>100,000	1: >1,000
G-361	Melanoma	63	5,100	1: 81
TFK-1	Cholangiocarcinoma	66	>100,000	1: >1,500
MOLT-3	T cell acute lymphoblastic leukemia	66	>100,000	1: >1,700
MOLT-4	T cell acute lymphoblastic leukemia	59	>100,000	1: >1,300
PEER	T cell acute lymphocytic leukemia	83	>100,000	1:>190
C1R	B-cell lymphoma	530	>100,000	1: >1,200
SCC-3	Non-Hodgkin’s lymphoma	81	>100,000	1: >1,700
Raji	Burkitt’s lymphoma	53	>100,000	1: >1,200
RAMOS-RA1	Burkitt’s lymphoma	140	>100,000	1: >1,500
U937	Acute monocytic leukemia	60	>100,000	1: >330
K562	Histiocytic lymphoma	80	>100,000	1: >1,900
THP-1	Erythroleukemia	300	>100,000	1: >700
HMC-1-8	Mammary carcinoma	920	>100,000	1:>110
YMB-1-E	Mammary carcinoma	630	>100,000	1: >160
MRK-nu-1	Mammary carcinoma	180	>100,000	1: >560

The concentrations of compounds **7** (pivoxil ester prodrug) and **14** (acid form) required to stimulate half maximal TNF-α secretion (EC_50_) from γδ Τ cells in response to various tumor cell lines incubated with **7** or **14** are shown.

### Inhibition of RAP1A-geranylgeranylation in tumor cells by compound 7

**Figure 4:**
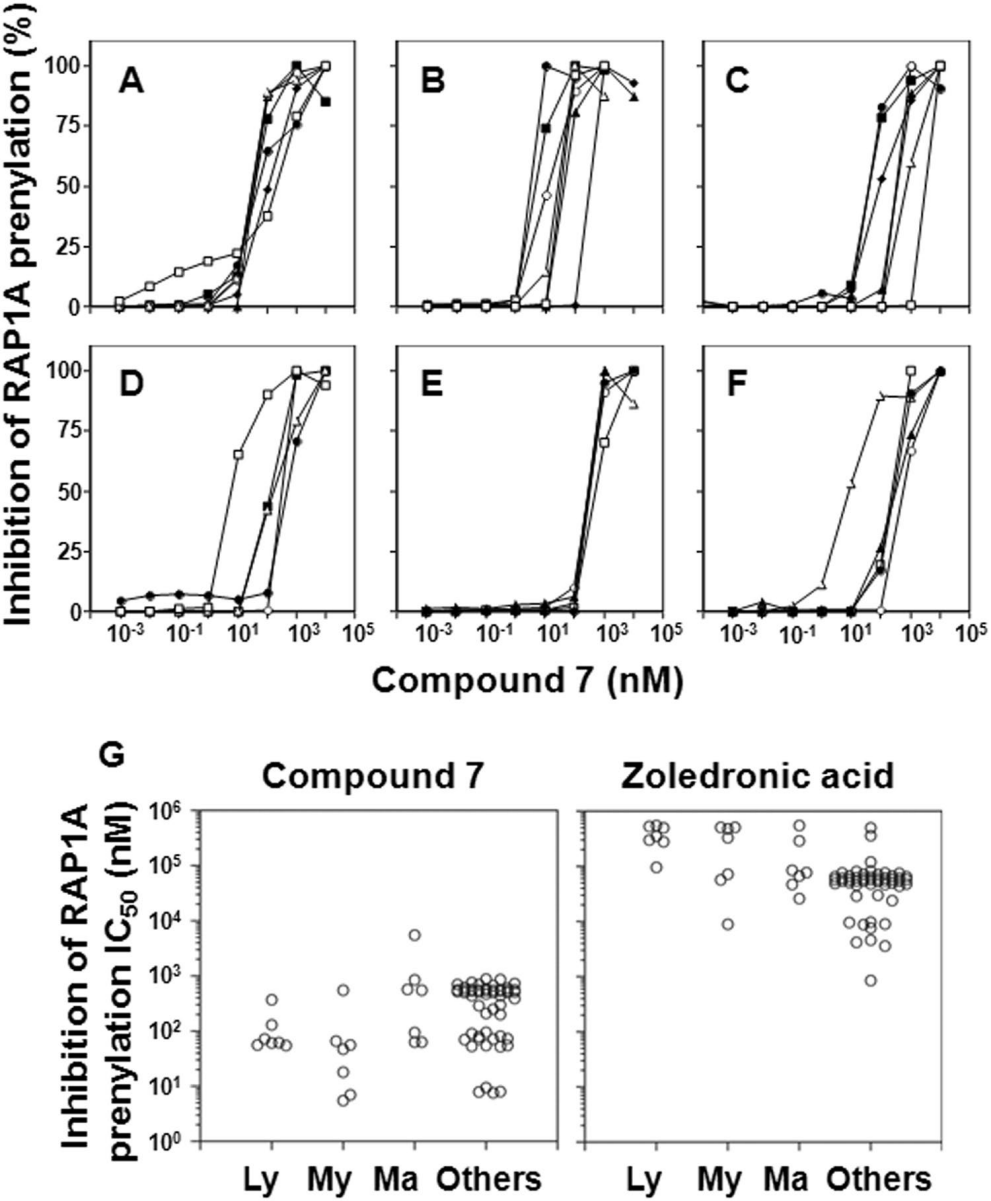
Inhibition of geranylgeranylation of RAP1A in tumor cells by compound **7**. Tumor cells were cultured with **7** for 16 h, lysed, and the prenylation of RAP1A assessed after protein separation by SDS-PAGE, transfer to PVDF membranes, and probing with anti-unprenylated RAP-1A antibodies. **(A**–**F**) Compound **7** inhibition of geranylgeranylation of RAP1A for various types of tumor cell lines: (**A**) lymphomas, ▫ MOLT-3, ∆ PEER, ⚪ C1R, ⬧ J.RT3-T3.5, ▪ Raji, ▴ RAMOS-RA1, ⚫ MOLT-4; (**B**) myeloid leukemias, ▫ HL60, ∆ U937, ⚪ THP-1, ⬧ SCC-3, ▪ P31/FUJ, ▴ K562, ⚫ NOMO-1; (**C**) mammary carcinomas, ▫ YMB-1-E, ∆ MRK-nu-1, ⚪ HMC-1-8, ⬧ MCF-7, ▪ MDA-MB-231, ▴ T-47D, ⚫ SK-BR-3; (**D**) renal cell carcinomas, ▴ 786-0, ∆ VMRC-RCZ, ⚫ UOK121, ⚪ Caki-1, ▪ A-704; (**E**) pancreatic carcinomas, ⚫ BXPC-3, ▴ KP4-1, ⚪ KP4-2, ▫ KP4-3, ∆ MiaPaCa2; (**F**) other tumor cells, ⚫ TGB24TKB cholangiocell carcinoma, ▴ ACS gastric carcinoma, ⚪ MG63 osteocarcoma, ▫ LK-2 lung carcinoma, ∆ EJ-1 bladder carcinoma. (**G**) Comparison of compound **7** (left panel) with zoledronic acid (right panel) concentrations required for half-maximal inhibition of prenylation of RAP1A in tumor cells. Ly = lymphomas, My = myeloid leukemia, Ma = mammary carcinomas, and Others = other tumor cell lines. Data for zoledronic acid effects are from Idrees *et al*.^46^ and are included for comparison.

Bisphosphonates indirectly stimulate Vγ2Vδ2 T cells by inhibiting FDPS resulting in the intracellular accumulation of IPP, its upstream metabolite. The binding of IPP to the B30.2 domain of BTN3A1 alters cells in an undefined way to make them stimulatory for Vγ2Vδ2 T cells^7, 9^. Additionally, downstream metabolites such as geranylgeranyl diphosphate and farnesyl diphosphophate, are depleted leading to the accumulation of unprenylated RAP1A, a small G protein required for cellular adhesion. Therefore, the accumulation of unprenylated RAP1A is a measure of FDPS inhibition (Supplemental Fig. S4). To confirm that **7** uses the same mechanism of action as other bisphosphonates, the concentration of compound **7** required for half-maximal inhibition (IC_50_) of RAP1A prenylation was determined for a variety of tumor cell lines (Fig. 4 and Supplemental Table S3). **7** was highly active at inhibiting the prenylation of RAP1A with six cell lines with IC_50_ values < 10 nM, the P31/FUJ and MONO-1 monocyte-like leukemia cells, the EJ-1 and T24 bladder carcinoma cells, the 786-0 renal cell carcinoma cells, and the KATOIII gastric carcinoma cells. The proportion of tumor cell lines with an IC_50_ of 100 nM or less was 71.4% for lymphoma, 85.7% for myeloid leukemias, 42.9% for mammary carcinomas, and 32.7% for other tumor cell lines (Fig. 4). The average IC_50_ ± S.D. were similar to those noted for stimulating TNF-α release with values of 115 ± 115 nM for lymphomas, 107 ± 197 nM for myeloid leukemias, 1,099 ± 1,965 nM for mammary carcinomas, and 386 ± 259 nM for other tumor cell lines. Again, the values for zoledronic acid, were much higher than those for **7** (Fig. 4D), with an average IC_50_ ± S.D. of 372,286 ± 164,995 nM (3,233-fold greater than that of compound **7**) for lymphomas, 281,129 ± 229,049 nM (2,626-fold) for myeloid leukemias, 163,143 ± 191,842 nM (149-fold) for mammary carcinomas, and 64,249 ± 172,850 nM (166-fold) for other tumor cell lines^46^. Therefore, similar to the EC_50_ values noted for stimulating TNF-α release, **7** was a highly potent inhibitor of RAP1A prenylation. Given that most strong inhibitors of GGPS have mono- or diacyl- chains to occupy the farensyl and/or the geranyl sites^47–49^, the activity of **7** is likely due to inhibition of FDPS.

To assess the degree of correlation between compound **7** activities, the concentrations of compound **7** required for γδ T cell activation (EC_50_) were correlated with those for FDPS inhibition (IC_50_) or for tumor growth inhibition for each of the tumor cell lines (Supplemental Table S3). Only tumor cell growth inhibition and TNF-α release (r = 0.282, *p* = 0.016) were significantly correlated for compound **7** (Supplemental Fig. S5, top left panel). The concentrations of compound **7** required to stimulate production of TNF-α release were not significantly correlated with those required for RAP1A inhibition nor were tumor cell growth inhibition and RAP1A inhibition correlated. In contrast, zoledronic acid exhibited highly significant correlations for cell inhibition and TNF-α stimulation (r = 0.454, *p* < 0.0001), cell inhibition and RAP1A inhibition (r = 0.556, *p* < 0.0001), and TNF-α stimulation and RAP1A inhibition (r = 0.499, *p* < 0.0001). Also, **7** and zoledronic acid activities were not significantly correlated (Supplemental Fig. S5). Because the pivoxil esters must be removed to activate **7**, the lack of correlation between most activities of **7** could reflect differences in esterase expression in the tumor cell lines or other factors besides FDPS inhibition that determine its activity. The activity of zoledronic acid, in contrast, is likely dependent primarily on FDPS inhibition. Hence, the high correlation for the three activities measured.

### Specific stimulation of γδ T cells expressing Vγ2 Vδ2 TCRs by compound **7**

**Figure 5:**
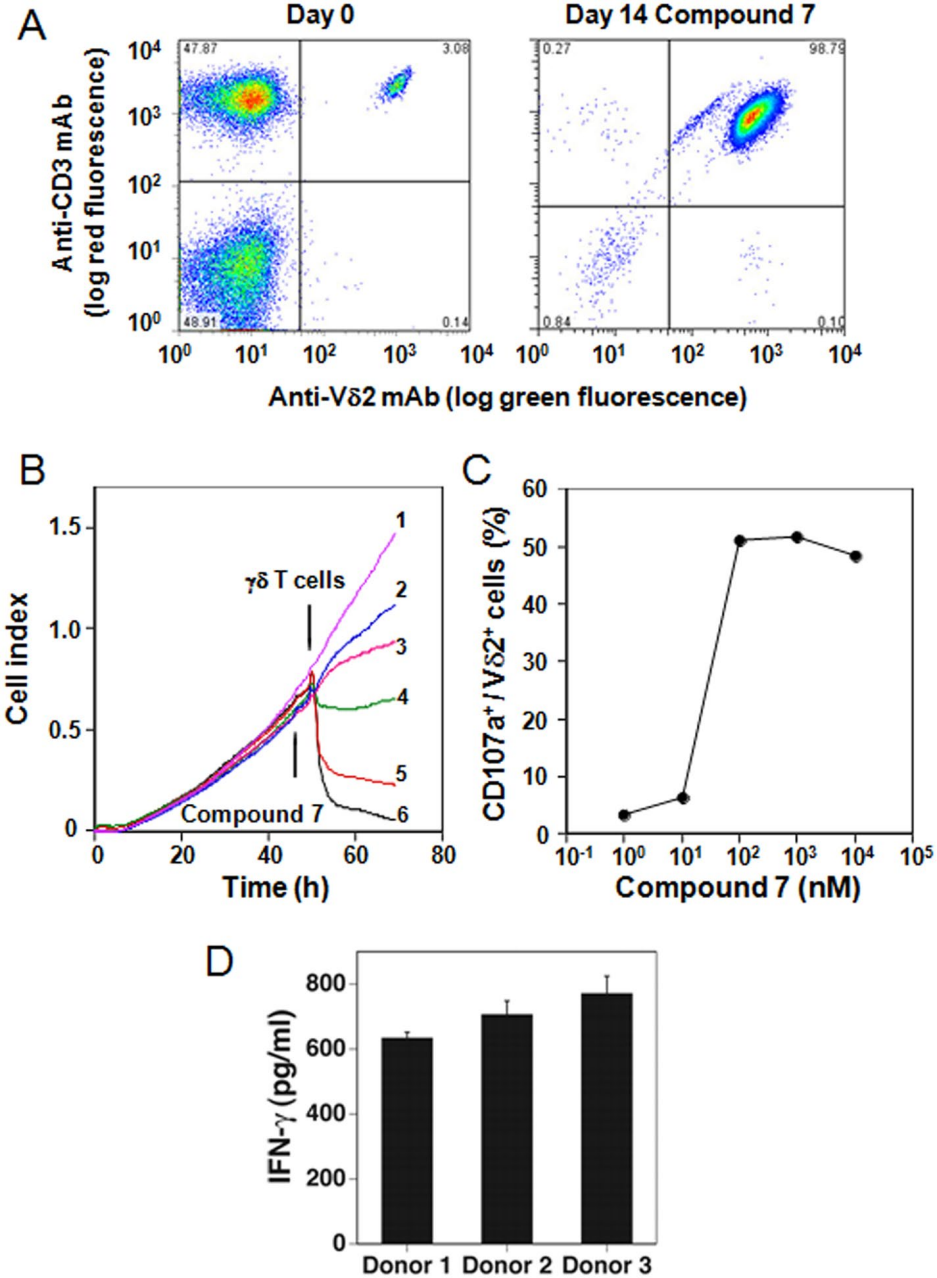
Selective activation of Vγ2Vδ2 T cells by compound **7**. (**A**) Selective expansion of Vγ2Vδ2 T cells from blood αβ and γδ T cells after culture with compound **7**. PBMC from a prostate cancer patient were stimulated with 1 μM compound **7** and IL-2. The two-color flow cytometric analysis of Vγ2Vδ2 T cells in PBMC before (left panel) and after (right panel) 10 day stimulation is shown. (**B**) Inhibition of EJ-1 bladder carcinoma cell growth by exposure to compound **7** and γδ T cells. Compound **7** was added to cultures of EJ-1 tumor cells followed by the addition of Vγ2Vδ2 T cell to some cultures16 h later. Cell growth was assessed in a real-time cell analyzer system. Culture conditions were: (1) 50 nM compound **7** + medium, (2) 0 nM compound **7** + γδ T cells, (3) 1.56 nM compound **7** + γδ T cells, (4) 12.5 nM compound **7** + γδ T cells, (5) 25 nM compound **7** + γδ T cells, (6) 50 nM compound **7** + γδ T cells. (**C**) Degranulation of γδ T cells in response to U937 histocytoma pretreated with compound **7**. The proportion of CD107a^+^ degranulated Vδ2^+^ cells were plotted against the concentrations of compound **7** used for the pretreatment of U937 cells. (**D**) Stimulation of IFN-γ production by Vγ2Vδ2 T cells by compound **7**. PBMC from healthy donor were cultured with 1 μM compound **7**. After 48 h, the culture supernatants were removed and IFN-γ levels determined by ELISA.

To assess the specificity of stimulation by compound **7**, PBMC were cultured with **7** and the levels of Vγ2Vδ2 T cells determined after 14 days. Despite the presence of other γδ and αβ T cells, only Vγ2Vδ2 T cells were selectively expanded to 98.8% of cells (Fig. 5A). The expansion of Vγ2Vδ2 T cells also demonstrates that compound **7** is not severely toxic to monocytes given that this response is dependent on their activity^50, 51^. To determine the specificity of compound **7** on cellular cytotoxicity of γδ T cells, their activity against T-47D mammary carcinoma cells was tested in a real-time cell growth assay. Exposure of T-47D mammary carcinoma cells to 50 nM of compound **7** did not affect their growth while exposure to γδ T cells slightly delayed their growth. In contrast, the addition of both compound **7** and γδ T cells to cultures of T-47D cells inhibited their growth in a compound **7** dose-dependent manner (Fig. 5B). Additionally, pretreatment of U937 histocytoma cells with compound **7** rendered them stimulatory for Vγ2Vδ2 T cell degranulation and surface expression of CD107a (Fig. 5C). Finally, compound **7** stimulated Vγ2Vδ2 T cells in PBMC to release interferon-γ (IFN-γ) (Fig. 5D). These results demonstrate that γδ T cells expressing Vγ2 Vδ2 TCRs are specifically activated to proliferate, kill tumor cells, and release IFN-γ by exposure to monocytes and tumor cells treated with **7**.

### Cancer therapy combining compound **7** and adoptive transfer of Vγ2Vδ2 T cells improves the survival of immunodeficient NOG mice inoculated with either human EJ-1 bladder carcinoma cells or HT1080 fibrosarcoma cells

**Figure 6:**
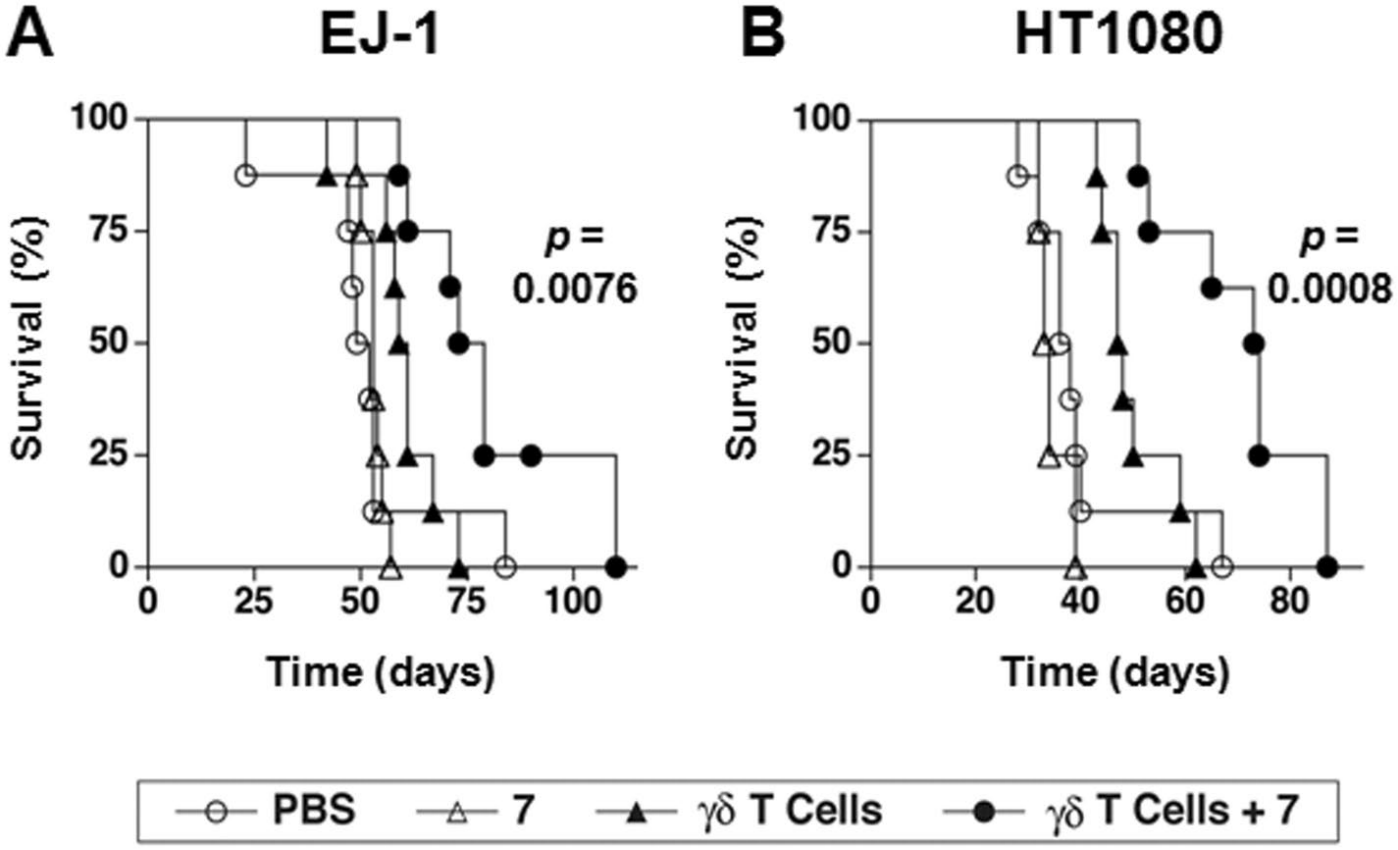
Combination therapy with compound **7** and γδ T cells prolongs survival of immunodeficient NOG mice inoculated with either human EJ-1 bladder carcinoma cells or human HT1080 fibrosarcoma cells. On day 0, NOG mice were inoculated i.p. with 1.0 × 10^6^ EJ-1 or 1.0 × 10^6^ HT1080 tumor cells that had been stably transfected with the *luc2* luciferase. On day 3 and day 6, the mice were treated with either (1) PBS (⚪), (2) compound 7 (∆) (2 μg (97 μg/kg) for EJ-1 or 10 μg (485 μg/kg) for HT1080), (3) 2 × 10^7^ γδ T cells (▴), or (4) 2 × 10^7^ γδ T cells and compound 7 (⚫) (2 μg (97 μg/kg) for EJ-1 or 10 μg (485 μg/kg) for HT1080). This treatment regimen was repeated for the duration of the experiment. (**A**) Survival of mice inoculated with EJ-1 cancer cells untreated or treated as above. Pooled data from two experiments performed identically is shown, n = 8 mice per group. (**B**) Survival of mice inoculated with HT1080 cancer cells untreated or treated as above. Pooled data from two experiments performed identically is shown, n = 8 mice per group except for mice treated with compound 7 where 4 mice were used. γδ T cells expressing Vγ2 Vδ2 TCRs (95–98% of transferred cells) were expanded from PBMC from a single donor and frozen for later use. Significance is shown for the survival difference between γδ T cells and compound 7 compared with γδ T cells alone as determined by the log-rank test.

Because BTN3A1 proteins and IPP-responsive γδ T cells are not present in mice, rats, guinea pigs, or rabbits, adoptive transfer models for human γδ T cell immunotherapy were developed. Immunodeficient NOG (NOD.Cg-*Prkdc* ^*scid*^ *Il2rg* ^*tm1Sug*^/Jic) mice were intraperitoneally inoculated with either human EJ-1 bladder carcinoma cells or human HT1080 fibrosarcoma cells. Tumor-bearing mice were then injected twice a week with either (1) PBS, (2) compound **7**, (3) γδ T cells, or (4) γδ T cells and compound **7** until mouse death or euthanasia. The Vγ2Vδ2 T cells were derived from a single donor by stimulation with zoledronic acid, frozen for use, and were between 95–98% pure (Supplemental Fig. S6). Mice inoculated with EJ-1 cells survived longer when treated with γδ T cells and compound **7** (median survival 76 days) than with γδ T cells alone (median survival 60 days) (Fig. 6A, *p* = 0.0076). No increase in survival was noted after treatment with **7** compared with control mice (median survival 53 days versus 50.5 days for PBS, *p* = 0.4348). Similar results were noted with a second solid tumor, the HT1080 fibrosarcoma. Mice treated with γδ T cells and compound **7** survived longer (median survival 73.5 days) than mice treated with γδ T cells alone (median survival 47.5 days) (Fig. 6B, *p* = 0.0008). Treatment with compound **7** alone did not prolong mouse survival (median survival 33.5 days versus 37 days for PBS, *p* = 0.2879). **7** was well tolerated with no obvious morbidity and no mortality of mice given a 3–15-fold higher doses than those used in these experiments (Supplemental Fig. S7). These results show that combined treatment with γδ T cells and compound **7** improves survival of mice bearing two different human tumors and that a variety of tumors would be potentially responsive to treatment.

## Discussion

Adoptive immunotherapy with Vγ2Vδ2 T cells for cancer has proven to be safe but of limited effectiveness. Treatment has resulted in partial and complete remissions and stable disease but most patients progress. In this study, we describe a new approach to therapy with Vγ2Vδ2 T cells by using bisphosphonate prodrugs where the phosphonate moiety is masked with pivoxil groups. Masking the phosphonate moiety greatly increases the potency of bisphosphonate stimulation of Vγ2Vδ2 T cells with several active at less than 100 nM. A thaizole compound, **7** (tetrakis-pivaloyloxymethyl 2-(thiazole-2-ylamino)ethylidene-1,1- bisphosphonate), was particularly active, stimulating TNF-α release by Vγ2Vδ2 T cells at concentrations as low as 40 nM. **7** was significantly more potent than zoledronic acid at stimulating Vγ2Vδ2 T cells with an average EC_50_ that is 909-fold lower than zoledronic acid. Consistent with FDPS inhibition as its mechanism of action, **7** exhibited potent inhibitory activity for RAP1A prenylation in tumor cells (3,200-fold more active than zoledronic acid) and tumor cell growth^43^ (184-fold more potent than zoledronic acid). Like other FDPS inhibitors, **7** selectively stimulated Vγ2Vδ2 T cells to proliferate, secrete cytokines, and kill tumor cells treated with **7**. Finally, combined therapy with **7** and Vγ2Vδ2 T cells extended the survival of immunodeficient mice inoculated with either human bladder carcinoma or fibrosarcoma cells. Taken together, our results suggest that the bisphosphonate prodrug, **7**, could significantly improve the efficacy of adoptive immunotherapy with Vγ2Vδ2 T cells.

To improve the activity of bisphosphonates, we designed and synthesized a series of bisphosphonate prodrugs^43^. In the present study, we have assessed bisphosphonate prodrug activation of Vγ2Vδ2 T cells and inhibition of RAP1A prenylation in response to a variety of human cancer cell lines pretreated with the compounds. Masking the negative charges of the phosphonate groups of bisphosphonates with pivoxil esters greatly increased their activity. However, biological activity varied between tumor cell lines. These variations could be explained by differences in the levels of intracellular esterase activity and/or in the substrate specificity of the esterases^52, 53^. Additionally, differences in the levels of FDPS and which oncogenes were driving tumor cell proliferation and function could also play a role in determining their sensitivity to bisphosphonate prodrugs. Compound **7**, a thiazole bisphosphonate prodrug, was highly active against most tumor cell lines. Hematopoietic cell lines were particularly sensitive to **7** averaging 796-fold more sensitive to **7** than zoledronic acid for growth inhibition, 3,487-fold more active for TNF-α release by γδ T cells than zoledronic acid, and 12,869-fold more inhibitory for RAP1A prenylation than zoledronic acid. Thus, the bisphosphonate prodrug **7** could be particularly effective for the treatment of lymphomas and myeloid leukemias.

One of the most important features of bisphosphonate prodrugs is their high membrane permeability that enables them to enter into tumor cells that have poor fluid-phase endocytosis. Because they were developed as anti-resorptive therapeutics, conventional bisphosphonates interact with hydroxyapatide crystals of bone with high affinity. In contrast, because of their hydrophobicity, bisphosphonate prodrugs will likely have low affinity for bone and, instead, distribute to non-skeletal tissues including tumors. The high membrane permeability and non-skeletal distribution of bisphosphonate prodrugs are ideal for their use in cancer immunotherapy targeting γδ T cells.

Clinical trials using adoptive immunotherapy with Vγ2Vδ2 T cells to treat various cancers have not routinely used bisphosphonate treatments to increase tumor immunogenicity^27–36^. However, the adoptive immunotherapy trials reporting the best results have also used zoledronic acid treatment. A complete remission in a patient with metastatic renal cell carcinoma (out of eleven patients treated) was achieved by treatment with intravenous zoledronic acid and IL-2 followed by adoptive transfer of Vγ2Vδ2 T cells on the same day and then intravenous IL-2 given for four consecutive days repeated monthly for six cycles^54^. In a second trial, there were no responses noted in patients treated with zoledronic acid and γδ T cells alone but one complete remission was achieved in a breast cancer patient treated with a combination of zoledronic acid and adoptive transfer of Vγ2Vδ2 T cells along with the continuation of hormonal therapy^30^. Partial or complete responses were also noted in patients with various solid tumors when chemotherapy was combined with the adoptive transfer of Vγ2Vδ2 T cells with^30^ or without^35^ zoledronic acid. Although stable disease was achieved in a number of patients with lung cancer^55^ and other solid tumors^31, 35, 36^, the most common response to the adoptive transfer of γδ T cells alone was progressive disease. Therefore, the combination of a nitrogen-containing bisphosphonate and γδ T cells appears more effective than either alone.

Combining bisphosphonate prodrugs with γδ T cells could significantly advance the use of γδ T cells for cancer immunotherapy for a wide range of tumors. Given the potency of compound **7** both for stimulating γδ T cells and for inhibiting tumor cell growth and its likely lowered bone binding and increased entry into rapidly metabolizing tumor cells compared with zoledronic acid, combination therapy with **7** and the adoptive transfer of γδ T cells could increase treatment efficacy. As shown in our study, Vγ2Vδ2 T cells respond to essentially all tumors after treatment with compound **7** irrespective of the number of mutations the tumor harbors. Unlike TIL or CAR-T therapy, successful Vγ2Vδ2 T cell adoptive transfer does not require preconditioning with chemotherapy or irradiation. Therefore, therapy with **7** and γδ T cells could be further combined with checkpoint blockade with anti-PD-1/anti-PD-L1 and/or anti-CTLA-4 antibodies to activate anti-tumor CD4 and CD8 αβ T cells that are already present. Other cancer immunotherapies such as indoleamine 2,3-dioxygenase inhibitors^56–58^ or COX1/2 blockers to inhibit prostaglandin E_2_ production^59^ could also be added. In summary, combining the bisphosphonate prodrug **7** and γδ T cells with other cancer immunotherapies could allow the treatment of a wide range of adult and pediatric solid tumors that are not presently amendable to successful chemotherapy or targeted therapy.

## Materials and Methods

### Synthesis of bisphosphonate prodrugs and their corresponding acid forms

The synthesis and chemical characterization of the bisphosphonate prodrugs and their acids used in these studies are described^43^.

### Expansion of Vγ2Vδ2 T cells

Peripheral blood samples were obtained after approval of the institutional review board of Kyoto University Hospital and with written informed consent from all participants. All methods were performed in accordance with the relevant guidelines and regulations of Kyoto University Hospital. PBMCs were purified by Ficoll-Paque^TM^ PLUS (GE Healthcare Bio-Sciences AB, Uppsala, Sweden) gradient centrifugation. The cells were washed two times with PBS, then resuspended in modified Yssel’s medium supplemented with 10% human AB serum (Cosmobio Co., Ltd., Koto-ku, Tokyo, Japan). They were cultured for 10 days at 2.5 × 10^6^ cells/1.5 ml in modified Yssel’s medium with 5 μM zoledronic acid and 100 IU/ml IL-2 (Shionogi Pharmaceutical Co., Ltd., Chuo-ku, Osaka, Japan) in a 24-well plate (Corning Inc., Corning, NY, USA). The culture medium was replaced every day from day 2 with fresh medium containing IL-2. The resulting expanded Vγ2Vδ2 T cells were then stored in liquid nitrogen for use upon thawing in *in vitro* assays or adoptive immunotherapy.

### TNF-α release by Vγ2Vδ2 T cells

Tumor cell lines were grown, harvested, and resuspended at 1 × 10^6^ cells/0.5 ml in 10-fold serial dilutions of BP prodrugs or acid forms of BPs in complete RPMI 1640 media (Sigma, St. Louis, MO, USA) supplemented with 10% fetal calf serum (Sigma), 10^−5^ M 2-mercaptoethanol (Nacalai Tesque, Kyoto, Japan), 100 IU/ml penicillin (Meiji Seika Kaisha, Tokyo, Japan), and 100 μg/ml streptomycin (Meiji Seika Kaisha). After incubation at 37 °C with 5% CO_2_ for 4 h, the cells were washed three times with 5 ml of the medium and resuspended in 0.5 ml of the same medium. A total of 0.1 ml (2 × 10^5^ cells/well) of the tumor cell suspension was placed on flat-bottomed 96-well plates and 0.1 ml of γδ T cells (2 × 10^5^ cells/well) was added. The plates were incubated at 37 °C with 5% CO_2_ for 16 h and the culture supernatants stored overnight at −80 °C. The samples were then thawed and TNF-α concentrations determined by ELISA (Peprotech, Rocky Hill, NJ, USA) using an ARVO spectrophotometer (PerkinElmer, Foster City, CA, USA). All experiments were performed in triplicate. Because bisphosphonic acids are not efficiently internalized into some tumor cells and were sometimes cytotoxic to tumor cells at 0.1 mM or greater, it was difficult to calculate EC_50_ values for some cell lines. In these cases, the value at 0.1 mM was considered to be the maximum value and used to calculate arbitrary EC_50_ values.

### Inhibition of geranylgeranylation of RAP1A in tumor cells

Tumor cells were resuspended in 90 ml of complete RPMI 1640 medium supplemented with 10% fetal bovine serum (FBS, Sigma), 10^−5^ M 2-mercaptoethanol (Invitrogen, Carlsbad, CA, USA), 100 IU/ml of penicillin (Meiji Seika Kaisha, Ltd., Chuo-Ku, Tokyo, Japan), and 100 μg/ml of streptomycin (Meiji Seika Kaisha) and grown overnight at 37 °C with 5% CO_2_ in 225 cm^2^ flasks. Compound **7** was then added to the flasks to the concentrations indicated above. After incubation for 16 h, the cells were harvested and resuspended in 100 μl of lysis solution containing 1% NP-40 (Wako Pure Chemical Industries Ltd., Chuo-ku, Osaka, Japan), 0.1% sodium dodecyl sulfate (SDS) (Tokyo Chemistry Industry Co., Ltd., Chuo-Ku, Tokyo, Japan), and 0.5% sodium deoxycholate (Wako) in microcentrifuge tubes. After centrifugation at 15,000 rpm for 10 min, the supernatants were transferred to new tubes and SDS-urea buffer containing 6.7 M urea (Wako), 5% SDS, 100 mM Tris–HCl buffer, pH 7.4 (Wako), 0.25% bromophenol blue (Wako), and 50 mM dithiothreitol (Wako) was added to give a protein concentration of 5 mg/ml. The samples were loaded on 15% polyacrylamide slab gels (Daiichi Pure Chemicals Co., Ltd., Chuo-ku, Tokyo, Japan) at 50 μg/lane, and electrophoresed at 120 mA/h. The proteins were then transferred onto Polyscreen (R) PVDF Transfer Membranes (PerkinElmer Inc., Waltham, MA) treated with goat anti-unprenylated RAP1A mAb (1 to 500 dilution, Santa Cruz Biotechnology Inc., Santa Cruz, CA, USA), and horse radish peroxidase-conjugated anti-goat IgG mAb (1 to 5,000 dilution, KPL Inc., Gaithersburg, MD, USA), followed by SuperSignal West Pico Chemiluminescent Substrate (Thermo Scientific, Rockford, IL, USA). Although not shown, controls using goat anti-RAP1A and anti-GAPDH mAbs (Santa Cruz Biotechnology) were included in this study. Chemiluminescence was detected on Amersham Hyperfilm^TM^ MP (GE Healthcare Ltd., Little Chalfont, Buckinghamshire, UK) using a Fuji Medical Film Processor FPM100 (Fuji Film Co., Ltd., Ashigara, Kanagawa, Japan). The strength of the signal for each protein band was determined by the brightness of the corresponding part of the image scanned using a LAS-4000 Mini Luminescent Image Analyzer (Fuji Film Co., Ltd.). The dose-response curves are based on the digitalized data.

### γδ T cell activation assays

For expansion of Vγ2Vδ2 T cells, PBMC from healthy donors were cultured at 2.5 × 10^6^ cells/1.5 ml in modified Yssel’s medium in the presence of 1 μM compound **7** in a 24-well plate (Corning Inc., Corning, NY, USA). The culture medium was replaced on day 2 with fresh medium containing IL-2. The cells were passaged on days 7, 9, 10, 11, and 12 and IL-2 (100 IU/ml) was added to the culture medium every day beginning from day 1. On day 14, the cells were harvested and analyzed for the expression of CD3 and Vδ2 by flow cytometry. For measurement of interferon-γ (IFN-γ) release, PBMC from healthy donors were cultured at 2.5 × 10^6^ cells/1.5 ml in modified Yssel’s medium in the presence of 1 μM compound **7** at 37 °C with 5% CO_2_ in a 24-well plate (Corning Inc., Corning, NY, USA). After 24 h, 100 IU/ml IL-2 (Shionogi Pharmaceutical Co., Ltd., Chuo-ku, Osaka, Japan) was added to the culture medium. After an additional 24 h, the culture supernatants were removed and stored overnight at −80 °C. The samples were then thawed and IFN-γ levels determined by ELISA (Peprotech, Rocky Hill, NJ, USA). All experiments were performed in triplicate.

### Inhibition of ***in vitro*** tumor cell growth by compound **7** and Vγ2Vδ2 T cells

Inhibition of cell growth by compound **7** and Vγ2Vδ2 T cells was measured using a real-time cell analyzer (RTCA) system comprising a 96-well E-plate with impedance sensors. Tumor cells were cultured for 24 h, harvested using 0.25% trypsin-EDTA solution (Invitrogen Gibco), and plated into 96-well E-plates at a density of 3 × 10^3^ cells/100 μl/well. Impedance was monitored every 15 min for 46.5 h at 37 °C with 5% CO_2_. 50 μl of supernatant was then removed from, and 50 μl of a serial dilution of compound **7** was added to each well. The plates were returned to the RTCA unit and incubated at 37 °C with 5% CO_2_ for 3.5 h. After 50 μl of the supernatant was removed, 1 × 10^5^ Vγ2Vδ2 T cells/50 μl were added to each well. Impedance was monitored for an additional 15 h. Five independent replicate experiments were performed, with eight wells per treatment group used in each individual replicate experiment.

### CD107a degranulation assay

Vγ2Vδ2 T cells (2 × 10^5^ cells/well) were incubated with U937 cells (2 × 10^5^ cells/well) that had been treated with serial dilutions of compound **7** for 2 h at 37 °C in 50 μl of complete RPMI 1640 medium in a 96-well culture plate in the presence of 5 μl of phycoerythrin-conjugated anti-CD107a mAb (BioLegend, San Diego, CA, USA) at 37 °C with 5% CO_2_. After 4 h of incubation, the cells were stained with 50 μl of 25-fold diluted FITC-conjugated anti-Vδ2 mAb fluorescein isothiocyanate-conjugated anti-TCR-Vδ2 mAb (Immunotech, Prague, Czech Republic) and analyzed using a FACSCalibur flow cytometer. The proportion of CD107a^+^ cells in Vδ2^+^ cells was plotted against concentrations of compound **7**.

### Inhibition of tumor cell growth by treatment with compound **7** and γδ T cells in NOG immunodeficient mice

Immunodeficient NOG (NOD.Cg-*Prkdc* ^*scid*^ *Il2rg* ^*tm1Sug*^/Jic) female mice 7–8 weeks of age were intraperitoneally (i.p.) inoculated with either 1.0 × 10^6^ EJ-1 bladder cancer cells or 1.0 × 10^6^ HT1080 fibrosarcoma cells that had been stably transfected with pGL4.10[luc2] (Promega) to express the *luc2* luciferase protein. On day 3 and 6, tumor-bearing mice were i.p. injected with either (1) 0.1 ml of PBS, (2) 2 μg (for EJ-1) or 10 μg (for HT1080) compound **7**, (3) 2 × 10^7^ γδ T cells, or (4) 2 × 10^7^ γδ T cells plus 2 μg (for EJ-1) or 10 μg (for HT1080) compound **7**. A dose of 2 μg of compound **7** corresponds to 97 μg/kg whereas 10 μg corresponds to 485 μg/kg. These treatment regimens were repeated for the duration of the experiment. Survival of the mice was recorded and plotted using Prism version 4.0 c. Vγ2Vδ2 T cells were expanded for 10 days from PBMC from a single donor with breast cancer as described above and frozen for use. Purity of the Vγ2Vδ2 T cells was between 95–98%. Animal use was approved by the institutional review board of Kyoto University Medical School. All methods were performed in accordance with the relevant guidelines and regulations of Kyoto University Medical School.

### Statistical analyses

The statistical significance of survival data was assessed using the log-rank test. The *p* values are shown in the figure. The Pearson correlation coefficient was determined by correlating the different biological activities of compound **7** and zoledronic acid. *p* < 0.05 was considered significant. Statistical analyses were done in Prism version 4.0c (GraphPad Software, La Jolla, CA).

## Electronic supplementary material

Supplementary Information
