# Supplementary material for: Anti-Tumor Activity and Immunotherapeutic Potential of a Bisphosphonate Prodrug
Source: Sci Rep. 2017 Jul 20;7:5987. doi: 10.1038/s41598-017-05553-0 (PMC5519590; doi:10.1038/s41598-017-05553-0)
Supplement: Supplementary file 1 — Supplementary Information [file 41598_2017_5553_MOESM1_ESM.pdf]

Supporting Information

Anti-Tumor Activity and Immunotherapeutic Potential of a Bisphosphonate Prodrug

Yoshimasa Tanaka*, Masashi Iwasaki, Kaoru Murata-Hirai, Kenji Matsumoto, Kosuke Hayashi, Haruki Okamura, Tomoharu Sugie, Nagahiro Minato, Craig T. Morita and Masakazu Toi

Y. Tanaka, M. Iwasaki, K. Murata-Hirai, K. Matsumoto, K. Hayashi
Center for Innovation in Immunoregulative Technology and Therapeutics, Graduate School of Medicine, Kyoto University, Kyoto, Japan

H. Okamura
Department of Tumor Immunology and Cell Therapy, Hyogo College of Medicine, Nishinomiya, Japan

T. Sugie, M. Toi
Department of Surgery, Graduate School of Medicine, Kyoto University, Kyoto, Japan

N. Minato, Y. Tanaka
Department of Immunology and Cell Biology, Graduate School of Medicine, Kyoto University, Kyoto, Japan

C. T. Morita
Department of Internal Medicine and the Interdisciplinary Graduate Program in Immunology, University of Iowa Carver College of Medicine, Veterans Affairs Health Care System, Iowa City, IA U.S.A.

Y. Tanaka
Center for Bioinformatics and Molecular Medicine, Graduate School of Biomedical Sciences, Nagasaki University, Nagasaki, Japan.

*Correspondence: Dr. Yoshimasa Tanaka, 1-12-4 Sakamoto, Nagasaki 852-8523, Japan, Email: ystanaka@nagasaki-u.ac.jp

Supplemental Table S1. Comparison of TNF-α secretion from γδ T cells in response to EJ-1 tumor cells pretreated with pivoxil ester prodrugs and their corresponding acids[1]

PE : H #	TNF-α EC_{50} (nM)		Ratio
	PE	H	PE EC_{50}:H EC_{50}
1 : 8	550	>100,000	1 : >180
2 : 9	4,500	>100,000	1 : > 20
3 : 10	480	48,000	1 : 100
4 : 11	700	>100,000	1 : >140
5 : 12	69	49,000	1 : 710
6 : 13	80	48,000	1 : 600
7 : 14	60	68,000	1 : 1,100

[1]Concentrations of pivoxil ester prodrugs required to stimulate half maximal TNF-α secretion (EC_{50}) by γδ T cells in response to prodrug-pretreated EJ-1 tumor cells were compared with those with the corresponding acid forms: PE; pivoxil ester BP prodrugs and H; corresponding BP acids.

Supplemental Table S2. Stimulation of TNF-α secretion by γδ T cells with EJ-1 tumor cells pretreated by pivoxil ester prodrugs[1]

Compound	TNF-α EC_{50} (nM)	Compound	TNF-α EC_{50} (nM)
7	60	36	8,000
39	62	15	>10,000
34	63	17	>10,000
18	380	19	>10,000
31	1,600	21	>10,000
38	1,900	24	>10,000
23	3,400	25	>10,000
33	3,600	26	>10,000
20	3,700	29	>10,000
32	4,400	30	>10,000
27	4,600	35	>10,000
22	4,800	37	>10,000
16	5,100	40	>10,000
28	6,500	41	>10,000
42	7,100		

[1]Concentrations of pivoxil ester prodrugs required to stimulate half maximal TNF-α secretion (EC_{50}) by γδ T cells in response to prodrug-pretreated EJ-1 tumor cells.

Supplemental Table 3. 7 and zoledronic acid activity against various human tumor cell lines[1]

Tumor cell line	Cell lineage	Cell growth inhibition		TNF-α stimulation		RAP1A inhibition		Cell growth inhibition	TNF-α stimulation	RAP1A inhibition
		**7** IC_{50} (nM)	Zol IC_{50} (nM)	**7** EC_{50} (nM)	Zol EC_{50} (nM)	**7** IC_{50} (nM)	Zol IC_{50} (nM)	Fold Difference Zol/**7**	Fold Difference Zol/**7**	Fold Difference Zol/**7**
C1R	B cell lymphoma	78	19000	530	500000	56	300000	243.6	943.4	5357.1
RAMOS-RA1	Burkitt's lymphoma	110	100000	140	260000	61	530000	909.1	1857.1	8688.5
Daudi	Burkitt's lymphoma	750	240000	42	55000	75	9200	320.0	1309.5	122.7
Raji	Burkitt's lymphoma	1600	63000	53	270000	62	350000	39.4	5094.3	5645.2
RPMI8226	Multiple myeloma	37	3500	24	2300	32	37000	94.6	95.8	1156.3
HL60	Acute promyelocytic leukemia	81	460000	53	520000	56	510000	5679.0	9811.3	9107.1
NOMO-1	Acute myeloid leukenia	36	28000	45	54000	5.5	330000	777.8	1200.0	60000.0
SCC-3	Monocyte-like cell line	180	13000	81	72000	550	72000	72.2	888.9	130.9
THP-1	Acute monocytic leukemia	35	14000	300	440000	18	480000	400.0	1466.7	26666.7
U937	Histiocytic lymphoma (monocyte-like)	260	16000	60	460000	47	57000	61.5	7666.7	1212.8
P31/FUJ	Acute myeloid leukemia	130	65000	56	72000	7	510000	500.0	1285.7	72857.1
K562	Erythroleukemia	29	2900	80	66000	66	8900	100.0	825.0	134.8
J.RT3.-T3.5	T cell acute lymphoblastic leukemia	150	20000	170	430000	130	96000	133.3	2529.4	738.5
MOLT-3	T cell acute lymphoblastic leukemia	130	74000	66	530000	370	500000	569.2	8030.3	1351.4
MOLT-4	T cell acute lymphoblastic leukemia	160	71000	59	63000	72	550000	443.8	1067.8	7638.9
PEER	T cell acute lymphoblastic leukemia	23	55000	83	510000	55	280000	2391.3	6144.6	5090.9
HMC-1-8	Breast cancer	880	21000	920	200000	550	67000	23.9	217.4	121.8
MCF-7	Breast cancer	560	26000	510	66000	94	85000	46.4	129.4	904.3
MDA-MB-231	Breast cancer	1000	9400	600	63000	63	26000	9.4	105.0	412.7
MRK-nu-1	Breast cancer	740	19000	180	380000	850	47000	25.7	2111.1	55.3
SK-BR-3	Breast cancer	610	18000	340	30000	63	77000	29.5	88.2	1222.2
T-47-D	Breast cancer	350	17000	72	55000	570	290000	48.6	763.9	508.8
YMB-1-E	Breast cancer	950	89000	630	520000	5500	550000	93.7	825.4	100.0
786-0	Renal cell carcinoma	880	2900	65	50000	7.9	67000	3.3	769.2	8481.0
786-0W	Renal cell carcinoma	230	3200	69	54000	250	8800	13.9	782.6	35.2
A-704	Renal cell carcinoma	400	4600	720	51000	200	48000	11.5	70.8	240.0
ACHN	Renal cell carcinoma	320	6600	93	930	540	9600	20.6	10.0	17.8
Caki-1	Renal cell carcinoma	960	8100	120	47000	550	72000	8.4	391.7	130.9
UOK111	Renal cell carcinoma	1900	5100	500	6300	90	57000	2.7	12.6	633.3
UOK121	Renal cell carcinoma	970	1300	500	39000	700	46000	1.3	78.0	65.7
VMRC-RCW	Renal cell carcinoma	3100	13000	440	13000	510	500000	4.2	29.5	980.4
VMRC-RCZ	Renal cell carcinoma	300	3200	250	54000	290	48000	10.7	216.0	165.5
EJ-1	Bladder cancer	26	2000	60	5500	9.4	7600	21.3	91.7	808.5
T24	Bladder cancer	1100	2600	340	42000	8	4200	2.4	123.5	525.0
TGBC1TKB	Cholangiocarcinoma (Gallbladder)	660	9800	94	17000	53	51000	14.8	180.9	962.3
TCBC2TKB	Cholangiocarcinoma (Gallbladder)	2300	2800	85	36000	72	9700	1.2	423.5	134.7
TCBC24TKB	Cholangiocarcinoma (Gallbladder)	410	3700	430	30000	500	67000	9.0	69.8	134.0
HuCCT1	Cholangiocarcinoma	140	12000	67	54000	70	71000	85.7	806.0	1014.3
MZChA2	Cholangiocarcinoma (Gallbladder)	530	1100	620	80000	55	3600	2.1	129.0	65.5
TFK1	Cholangiocarcinoma	100	9500	66	52000	210	81000	95.0	787.9	385.7
ACS	Gastric cancer	270	3800	97	65000	550	50000	14.1	670.1	90.9
AGS	Gastric cancer	290	2700	79	71000	600	58000	9.3	898.7	96.7

GCIY	Gastric cancer	32	3900	70	6700	81	64000	121.9	95.7	790.1
KATOIII	Gastric cancer	142	3500	61	7900	7.6	62000	24.6	129.5	8157.9
MKN1	Gastric cancer	1200	12000	560	47000	560	61000	10.0	83.9	108.9
MKN28	Gastric cancer	540	17000	40	5600	670	65000	31.5	140.0	97.0
MKN45	Gastric cancer	45	4000					88.9		
MKN74	Gastric cancer	180	7800	88	7400	620	59000	43.3	84.1	95.2
ASPC1	Pancreatic cancer	260	14000	83	28000	880	75000	53.8	337.3	85.2
BXPC-3	Pancreatic cancer	140	4000	250	48000	570	55000	28.6	192.0	96.5
KP4-1	Pancreatic cancer	770	400	640	65000	510	62000	0.5	101.6	121.6
KP4-2	Pancreatic cancer	580	370	510	64000	540	57000	0.6	125.5	105.6
KP4-3	Pancreatic cancer	820	1200	430	65000	730	44000	1.5	151.2	60.3
MiaPaca2	Pancreatic cancer	150	370	560	55000	530	61000	2.5	98.2	115.1
Panc1	Pancreatic cancer	790	4000	570	65000	530	54000	5.1	114.0	101.9
PK1	Pancreatic cancer	340	15000	450	32000	500	58000	44.1	71.1	116.0
PK8	Pancreatic cancer	130	7700	290	54000	73	30000	59.2	186.2	411.0
PK9	Pancreatic cancer	270	7900	82	39000	500	54000	29.3	475.6	108.0
T3M4	Pancreatic cancer	270	8800	78	32000	390	60000	32.6	410.3	153.8
hu2	Liver cancer	260	18000	90	68000	590	65000	69.2	755.6	110.2
C32TG	Melanoma	4900	20000	59	6800	440	29000	4.1	115.3	65.9
G361	Melanoma	123	7100	63	7700	95	24000	57.7	122.2	252.6
Colo320	Colon cancer	390	21000	350	47000	470	76000	53.8	134.3	161.7
CW-2	Colon cancer	130	7400	770	510000	520	82000	56.9	662.3	157.7
DLD-1	Colon cancer	440	3400	64	6300	570	57000	7.7	98.4	100.0
GCT-IZ	Giant-cell tumor	2200	7000	700	50000	80	53000	3.2	71.4	662.5
HOS	Osteosarcoma	350	3900	68	23000	72	76000	11.1	338.2	1055.6
HuO	Osteosarcoma	2300	12000	570	61000	300	9000	5.2	107.0	30.0
MC3T3-E1	Osteosarcoma	590	4100					6.9		
MG67	Osteosarcoma	5000	8800	600	61000	760	54000	1.8	101.7	71.1
NY	Osteosarcoma	500	1400					2.8		
OST	Osteosarcoma	810	2500	600	370000	55	54000	3.1	616.7	981.8
SAOS2	Osteosarcoma	370	5400	86	6500	89	49000	14.6	75.6	550.6
TAKAO	Osteosarcoma	740	17000	510	54000	550	120000	23.0	105.9	218.2
LK-2	Lung cancer NSCLC Squamous	800	5800	97	26000	440	47000	7.3	268.0	106.8
SBC-2	Lung cancer SCLC	650	20000	74	13000	700	360000	30.8	175.7	514.3
PC-3	Prostate cancer	220	18000	65	6100	860	4600	81.8	93.8	5.3
HT1080	Fibrosarcoma	93	1200	80	9900	52	850	12.9	123.8	16.3

Mean	Hematopoietic tumors	237	77775	127	303357	104	288756	795.9	3486.5	12868.7
SD		403	117286	135	201007	146	211171	1423.7	3182.2	22027.4
Mean	Non-hematopoietic tumors	767	9570	298	68299	463	75982	26.5	297.4	577.6
SD		916	59710	240	158038	655	157633	28.9	353.2	1499.2
Mean	All tumors	658	23561	259	111119	386	121374	184.3	903.5	3199.7
SD		916	59710	240	158038	655	157633	702.4	1855.5	11216.5

[1] Data for compound **7** inhibition of the growth of tumor cell lines[1] and for zoledronate have been previously reported[2]. Averages for TNF-α release omit data from Daudi and RPMI8226 because they directly stimulate Vγ2Vδ2 T cells in the absence of bisphosphonates.

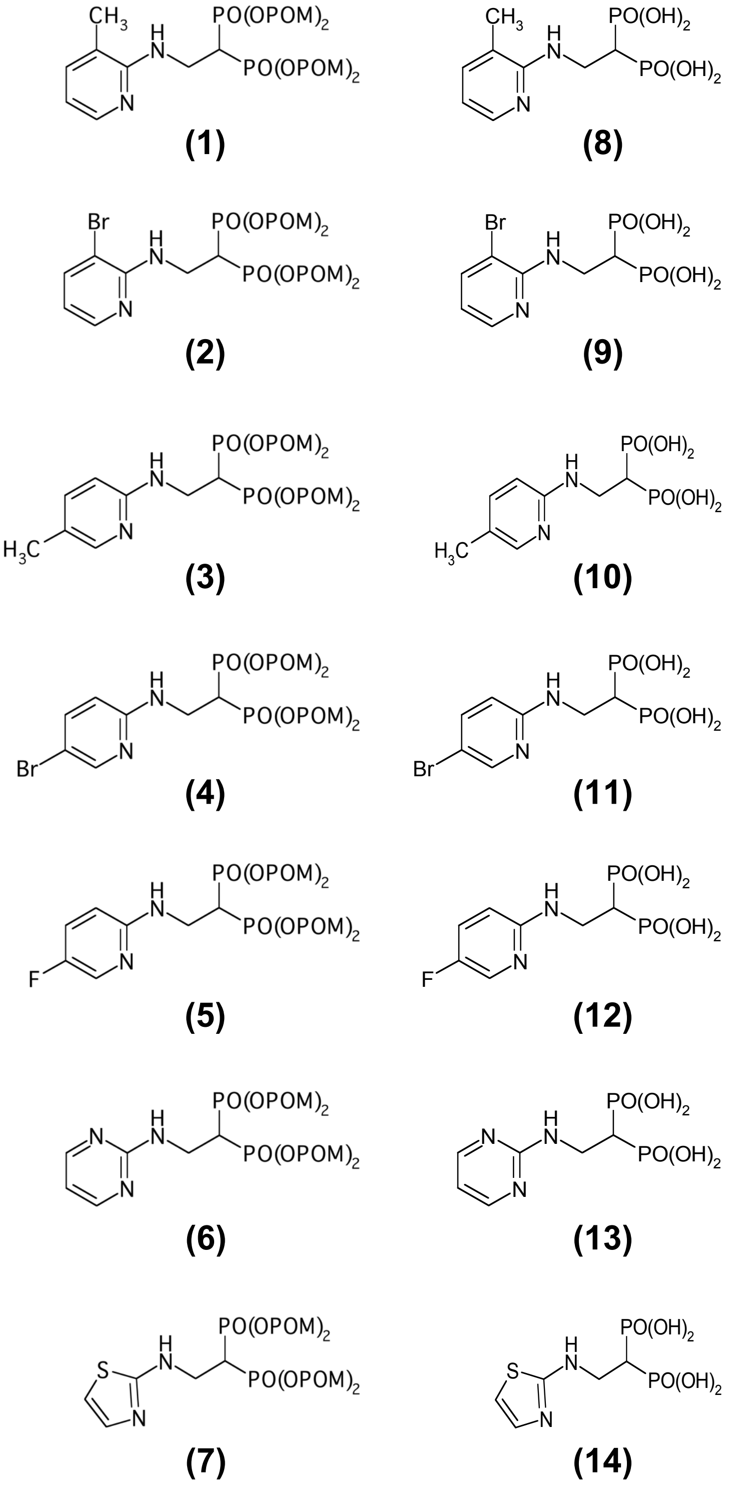

Supplemental Figure S1. Structures of pivoxil ester prodrugs and their acid forms. Pivoxil ester prodrugs (**1**-**7**) and their corresponding acids (**8**-**14**) are depicted. Details of their synthesis are previously described[1].

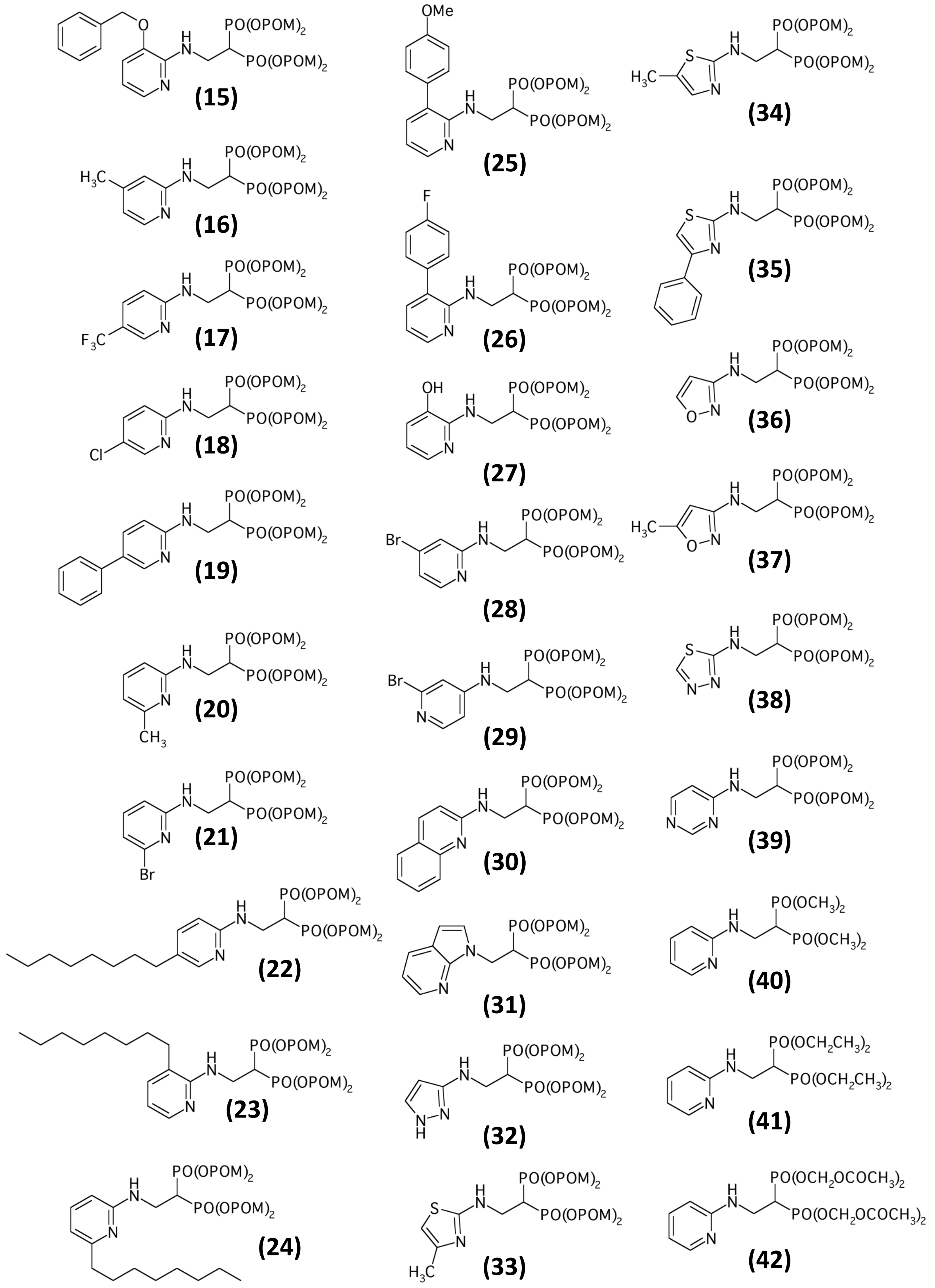

Supplemental Figure S2. Structures of pivoxil ester prodrugs (15-42). Details of their synthesis are previously described[1].

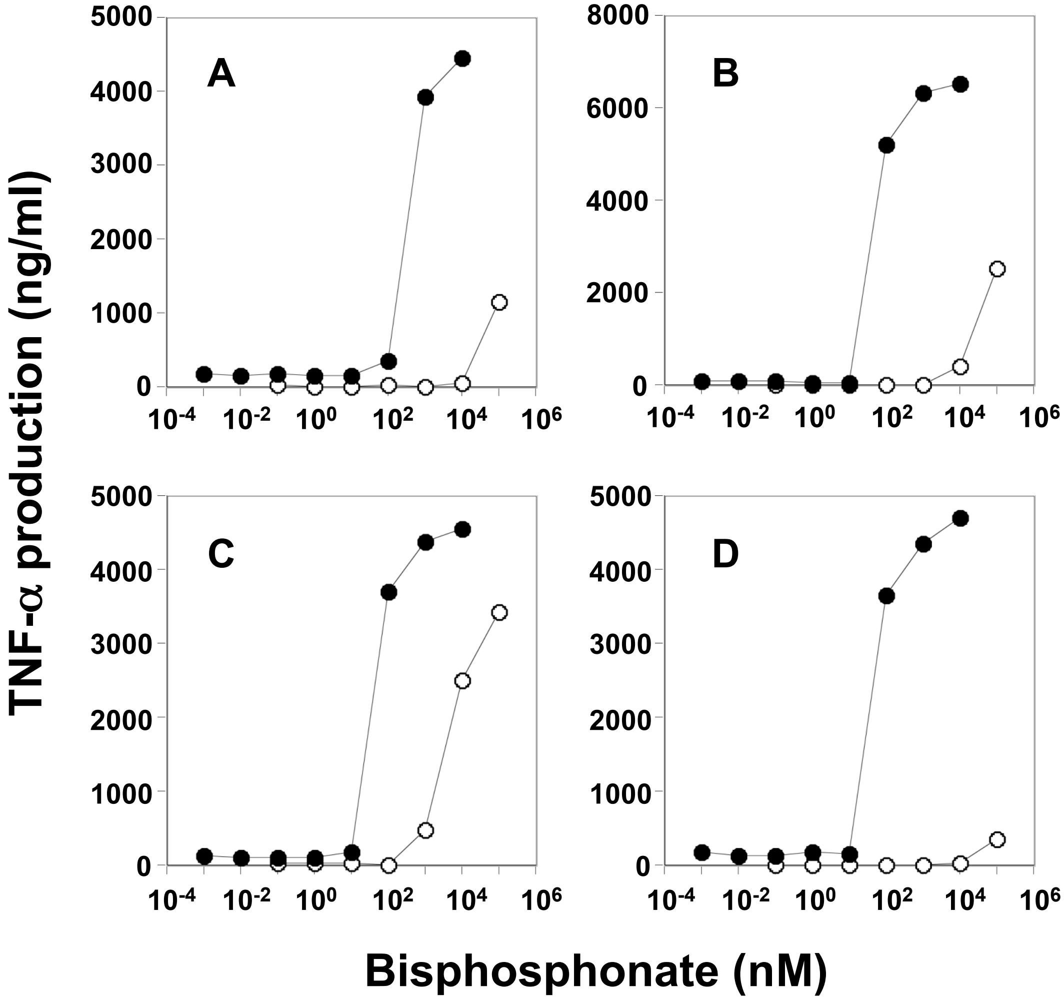

Supplemental Figure S3. Effect of pivoxil esterification on TNF-α secretion from γδ T cells stimulated with bisphosphonate-pretreated tumor cells. TNF-α production from γδ T cells in response to tumor cells pretreated with various concentrations of compound **7** (●) was compared with that of compound **14** (○): A; MKN1 gastric carcinoma, B; PC-3 prostatic carcinoma, C; G-361 melanoma, D; TFK-1 cholangiocell carcinoma.

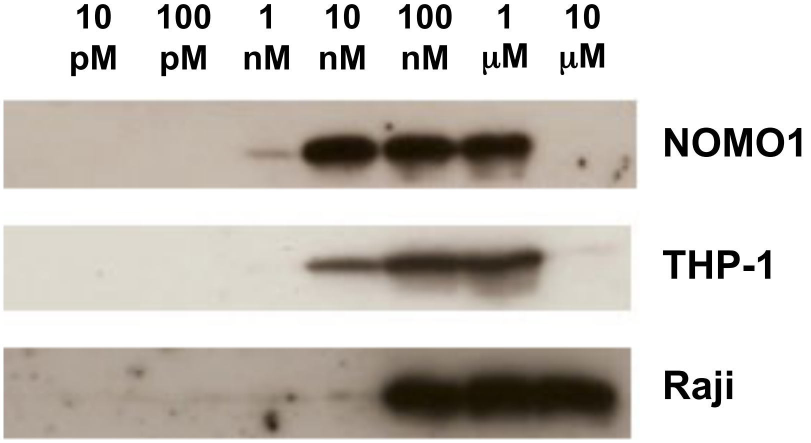

Supplemental Figure S4. Inhibition of geranylgeranylation of RAP1A in tumor cells by compound 7. Tumor cells were resuspended in 90 ml of complete RPMI 1640 medium supplemented with 10% fetal bovine serum (FBS, Sigma, St. Louis, MO), 10^{-5} M 2-mercaptoethanol (Invitrogen Corp., Carlsbad, CA), 100 IU/ml of penicillin (Meiji Seika Kaisha, Ltd., Chuo-Ku, Tokyo, Japan), and 100 μg/ml of streptomycin (Meiji Seika Kaisha) and grown overnight at 37°C with 5% CO_2 in 225 cm^2 flasks. Compound **7** was then added to the flasks to the concentrations indicated above. After incubation for 16 h, the cells were harvested and resuspended in 100 μl of lysis solution containing 1% NP-40 (Wako Pure Chemical Industries Ltd., Chuo-ku, Osaka, Japan), 0.1% sodium dodecyl sulfate (SDS) (Tokyo Chemistry Industry Co., Ltd., Chuo-Ku, Tokyo, Japan), and 0.5% sodium deoxycholate (Wako) in microcentrifuge tubes. After centrifugation at 15,000 rpm for 10 min, the supernatants were transferred to new tubes and SDS-urea buffer containing 6.7 M urea (Wako), 5% SDS (Tokyo Chemistry Industry), 100 mM Tris–HCl buffer, pH 7.4 (Wako), 0.25% bromophenol blue (Wako), and 50 mM dithiothreitol (Wako) were added to give a protein concentration of 5 mg/ml. The samples were loaded on 15% polyacrylamide slab gels (Daiichi Pure Chemicals Co., Ltd., Chuo-ku, Tokyo, Japan) at 50 μg/lane, and electrophoresed at 120 mA/h. The proteins were then transferred onto Polyscreen (R) PVDF Transfer Membranes (PerkinElmer Inc., Waltham, MA) treated with goat anti-unprenylated RAP1A Ab (1 to 500, Santa Cruz Biotechnology Inc., Santa Cruz, CA), and horse radish peroxidase-conjugated antigoat IgG Ab (1 to 5,000, KPL Inc., Gaithersburg, MD), followed by SuperSignal West Pico Chemiluminescent Substrate (Thermo Scientific, Rockford, IL). Although not shown, controls using goat anti-RAP1A and anti-GAPDH Abs (Santa Cruz Biotechnology) were included in this study. Chemiluminescence was detected on Amersham HyperfilmTM MP (GE Healthcare Ltd., Little Chalfont, Buckinghamshire, UK) using a Fuji Medical Film Processor FPM100 (Fuji Film Co., Ltd., Ashigara, Kanagawa, Japan). Of the 73 tumor cell lines tested, images from three representative cells are shown above. The strength of the signal for each protein band was determined by the brightness of the corresponding part of the image scanned using a LAS-4000 Mini Luminescent Image Analyzer (Fuji Film Co., Ltd.). The dose-dependency curves in Figure 4 are based on digitized data.

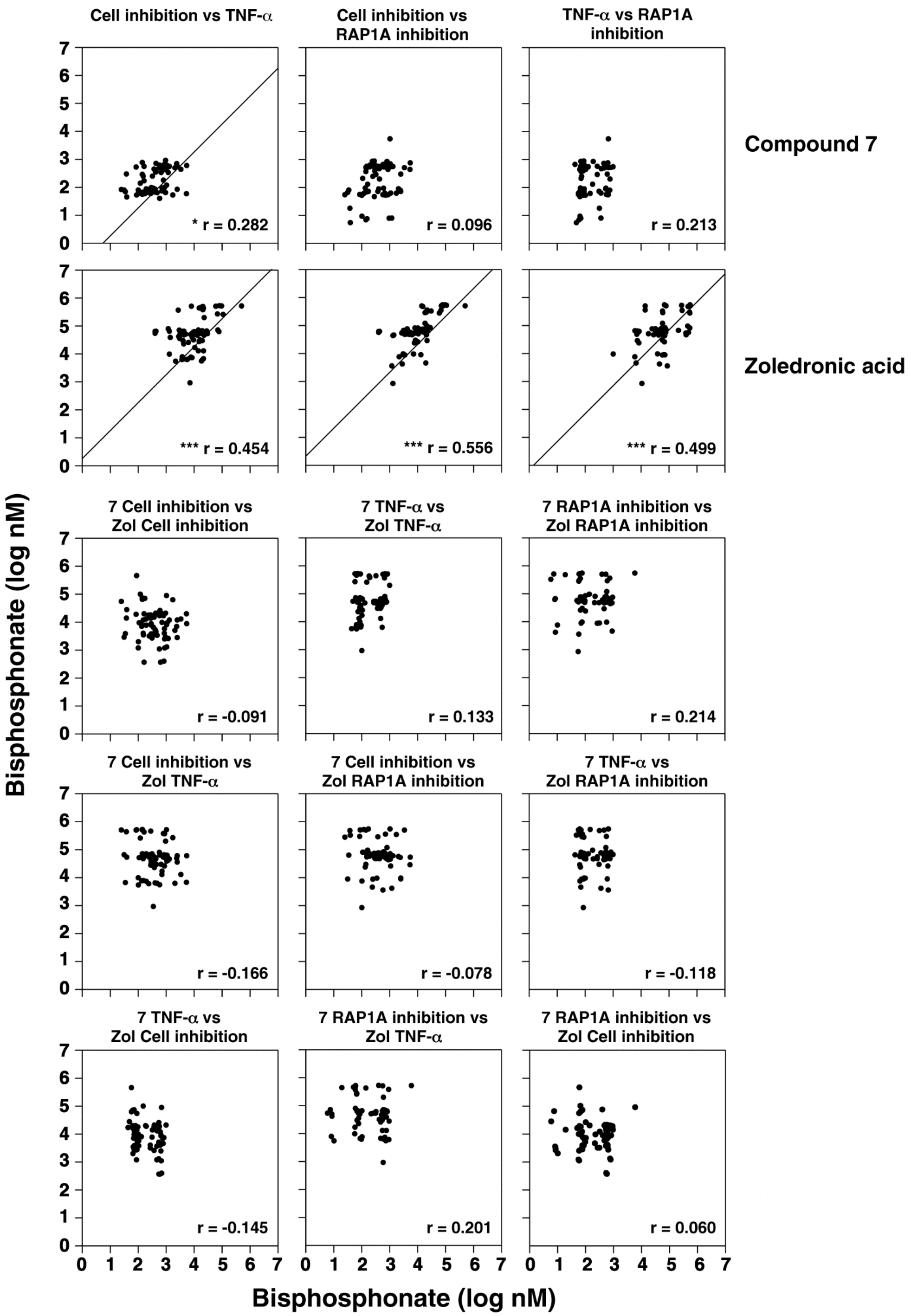

Supplemental Figure S5. Correlation between activity of compound 7 or zoledronic acid for γδ T cell activation, FDPS inhibition, and tumor cell growth inhibition. The correlations for compound **7**, zoledronic acid, and 7 / zoledronic acid concentrations required for half-maximal stimulation of TNF-α secretion by γδ T cells, half-maximal inhibition of RAP1A prenylation in tumor cells, and half-maximal inhibition of tumor cell growth are shown. The compound **7** tumor cell growth inhibition data and the zoledronic acid data were previously reported[1,2] and are shown for comparison. Data from Daudi and RPMI8226 are omitted from the analysis because they directly stimulate γδ T cells. r = Pearson's correlation coefficient. *$p < 0.02$ ***$p < 0.0001$

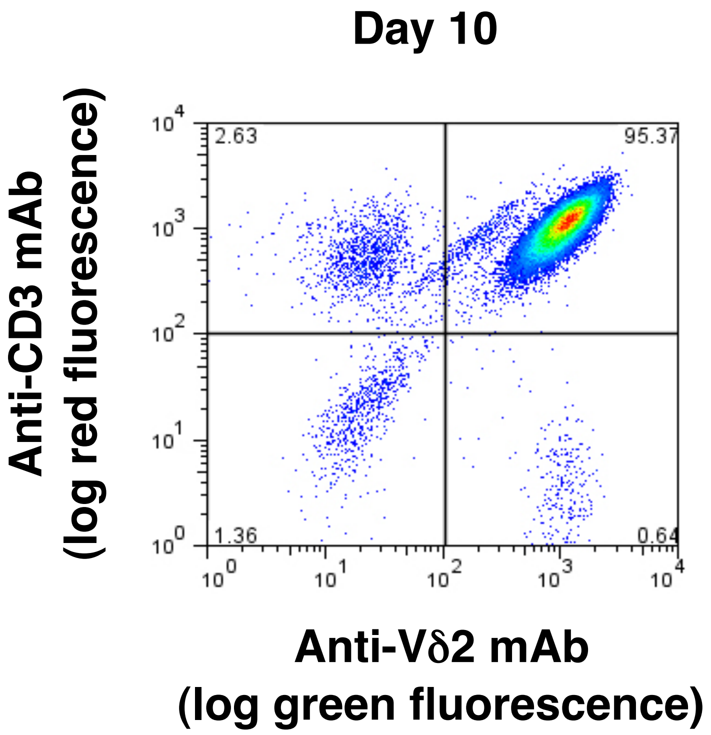

Supplemental Figure S6. Purity of γδ T cells expanded by zoledronic acid used in adoptive immunotherapy experiments in Figure 6 and 7. PBMC from normal donors was stimulated with zoledronic acid for 10 days as described. Expanded γδ T cells were washed, frozen, and then thawed to treat NOG mice inoculated with either EJ-1 or HT1080 tumor cell lines. Purity of γδ T cells was between 95-98%.

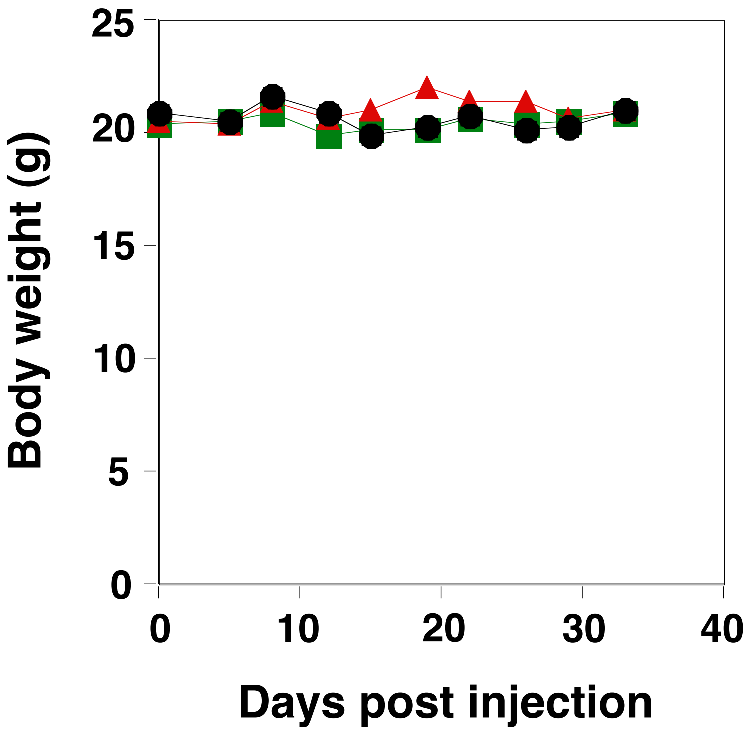

Supplemental Figure S7. Toxicity of compound 7 for mice. Three 8-week old female Balb/c mice were intraperitoneally injected with 30 μg of compound **7** and body weight was measured for 33 days after injection. Representative of two experiments.

References

1. Matsumoto, K. *et al*. Targeting cancer cells with a bisphosphonate prodrug. *ChemMedChem* **11**, 2656-2663 (2016).

2. Idrees, A.S. *et al*. Comparison of γδ T cell responses and farnesyl diphosphate synthase inhibition in tumor cells pretreated with zoledronic acid. *Cancer Sci* **104**, 536-542 (2013).
